# Supplementary material for: Monolithically-stacked thin-film solid-state batteries
Source: Commun Chem. 2023 Jun 5;6:110. doi: 10.1038/s42004-023-00901-w (PMC10241883; doi:10.1038/s42004-023-00901-w)
Supplement: Supplementary file 2 — Supplementary Information [file 42004_2023_901_MOESM2_ESM.pdf]

Supplementary Information for

Monolithically-stacked thin-film solid-state batteries

Moritz H. Futscher[‡,], Luc M. Brinkman[‡], André Müller,*

Joel Casella, Abdessalem Aribia, and Yaroslav E. Romanyuk[*]

[1] Laboratory for Thin Films and Photovoltaics, Empa - Swiss Federal Laboratories for Materials Science and Technology, Überlandstrasse 129, 8600 Dübendorf, Switzerland

[*] Corresponding author

[‡] These authors contributed equally

Supplementary Notes

Supplementary Note 1: Performance of individual electrodes

To evaluate the performance of the individual electrodes, we fabricated LCO/Lipon/Li and Si/Lipon/Li cells. Their voltage versus capacity curves is shown in Figure S1. The amorphous LCO cathode exhibited a substantial capacity fade within the first five cycles of 21% (Figure S1d), compared to 5% of the Si anode (Figure S1e). On the other hand, the multi-cell battery showed a capacity fade of 26% within the first 5 cycles (Figure S1f). Therefore, we conclude that the capacity fade of the amorphous LCO electrode is responsible for most of the capacity fade observed in our multi-cell battery, which we attribute to the amorphous nature of LCO. It should be noted that the stoichiometry of our amorphous LCO cathode is unknown. Further studies are required to address the question of how cycling-related volume changes affect the long-term performance of multi-cell batteries.

We note that additional capacity fading may come from the volume expansion of the Si anode, especially during prolonged cycling. However, the cathode loading per cell (4 $\mu Ah \cdot cm^{-2}$) is much lower than the anode loading (41 $\mu Ah \cdot cm^{-2}$). Therefore, we expect to observe a small volume change in the order of 10 nm within the Si anode.

Figure S2 shows the individual electrodes' differential capacity (dQ/dV) profiles in a single-cell configuration- with a Li-metal anode and the multi-cell battery, corresponding to the first five cycles at C/10. The differential capacity profiles of the amorphous LCO cell demonstrate one insertion and one desorption peak, which somewhat differ in position and shape from the crystalline LCO due to its amorphous nature (Figure S2a). In addition, the fast capacity fade is clearly visible. Si demonstrates two alloying and dealloying contributions: the alloying reaction of a-Si -> a-$Li_{2.0}Si$ -> a-$Li_{3.5}Si$ and the dealloying reaction of a-$Li_{3.5}Si$ -> a-$Li_{2.0}Si$ -> a-Si (Figure S2b).[2] The differential capacity profile of the multi-cell battery features broad insertion and desertion profiles indicative of amorphous LCO, superimposed with peaks that can be assigned to the alloying and dealloying reactions of Si (Figure S2c).

The Bragg-Brentano XRD diffractograms presented in Figure S3a confirm the absence of any crystalline phase in both as-deposited Si and LCO electrodes. Furthermore, grazing-incidence XRD measurements of LCO (Figure S3b) and Si (Figure S3c) also show no evidence of the LCO

(003) and Si (111) peaks, further verifying that no crystalline phase is present for the LCO cathode or the Si anode.

Supplementary Note 2: Pre-cycling of series-stacked thin-film battery

After fabrication of the series-stacked thin-film battery, each cell was individually pre-cycled with a C-rate of C/5 (2 $\mu A \cdot cm^{-2}$) to charge to 4.25 V and with a C-rate of C/10 (1 $\mu A \cdot cm^{-2}$) to discharge back to 3.0 V. During pre-cycling of one cell, the open-circuit voltage of the other cell was recorded. The pre-cycling of both cells is shown in Figure S4. It can be seen that cell 2 (top cell) changes its open-circuit voltage while cell 1 (bottom cell) is cycled (Figure S4a). This crosstalk between the two cells may be due to the Lipon bridge connecting the top and bottom cells (see Figure 2a in the main text). This crosstalk between the two cells is much less pronounced when the cell that is in open-circuit is already precycled (Figure S4b). However, in a future iteration of stacked thin-film batteries, such a Lipon bridge should be avoided. Since cell 2 had a higher capacity in the initial charge/discharge cycle than cell 1, we charged the voltage of cell 2 to 3.25 V before cycling both cells together. During the cycling of both cells, the voltage was controlled only by cell 1, which was cycled between 3.0 and 4.2 V while the current flowed through the whole stack. As a result, both cells are charged and discharged simultaneously.

Supplementary Methods

Supplementary Method 1: Lumped 0D steady-state thermo-electric model

The performance potential of a multi-cell thin-film battery was calculated using a lumped 0D steady-state thermo-electric model. The model calculates the specific energy and rate capability of multi-cell thin-film batteries, considering the operating limits imposed by voltage efficiency, critical current density, and thermal constraints. The model is agnostic regarding whether the cells are stacked in series or parallel. The assumptions of the model include the following:

i) steady-state (therefore no transient effects and no capacitances),

ii) 0D, i.e., a lumped model,

iii) no cell casing,

iv) an anode-free configuration,

v) one 4 µm thick Al substrate per battery,

vi) ten cells per battery,

vii) each cell consists of a 0.3 µm thick Al current collector, a 0.1 µm thick Cu current collector, a 1 µm thick Lipon solid electrolyte, and a variable thickness for the cathode,

viii) a battery width of 8 cm and an unlimited length, which is loosely based on the geometry of the BYD blade battery,

ix) a fixed voltage plateau at 3.7 V for both LCO and NMC811,

x) a capacity of 140 $mAh \cdot g^{-1}$ for LCO and 170 $mAh \cdot g^{-1}$ for NMC811,

xi) a limiting resistivity of 10'000 Ω·m for LCO and 3.3 Ω·m for NMC811,[4] and

xii) thermal conductivities of 239, 386, 3.7, 2, 7, and 1.7 $W \cdot m^{-1} \cdot K^{-1}$ for Al, Cu, LCO, NMC811, Lipon, and Si, respectively, and

xii) no effects of temperature on the behavior of the battery, such as changed conductivities.

The electrical model includes ten monolithically stacked cells on an Al substrate and lumps the electrochemical behavior of the cells into a constant voltage source in series with resistances representing the ionic and electronic conductivity of the different material layers. The voltage is calculated as

$$V = V_{OCV} - V_{loss} = V_{OCV} \cdot \eta_V \quad (1)$$

were η_V is the voltage efficiency and V_{loss} the voltage loss, which is a function of the current I times the sum of all the internal resistances

$$V_{loss} = I \ \cdot \Sigma_i R_i. \quad (2)$$

The internal resistance R_i of each layer is calculated as

$$R_i \ = \rho_i \ \cdot d_i \quad (3)$$

where ρ_i is the electronic or ionic resistivity of the layer and d is the layer thickness. For the current collectors, we use electronic resistivity, and for the electrolyte, we use ionic

resistance. Since ion and electron resistance plays a role in the cathode and the proportions depend on the state of charge, we assume the worst case, i.e., the greatest of the two resistances.

The power output of the battery is defined as:

$$P = V \cdot I = (V_{OCV} - I \cdot R_{cell}) \cdot I \tag{4}$$

The maximum power hence occurs at $V = 0.5 \cdot V_{OCV}$, as shown in Figure S9.

$$\frac{dP}{dI} = V_{OCV} - 2 \cdot I \cdot R_{cell} = 0 \Rightarrow I \cdot R_{cell} = V_{ocv}/2 \tag{5}$$

Thermal behavior is modeled by Joule heating and 1D steady-state heat conduction along the width of the battery, as schematically shown in Figure 3a in the main text. Here we assume heat generation within the battery and that that the temperature is independent of time. The 1D steady-state heat-diffusion equation is defined as

$$\frac{d^2T(x)}{dx^2} + \frac{\mathrm{q}}{k} = 0 \tag{6}$$

where T (K) is the temperature at position x in m within the cell, q (W·cm^{-3}) is the volumetric rate of internal heat generation, and k (W·m^{-1}·K^{-1}) is the thermal conductivity of the material. This second-order differential equation can be solved as

$$T = -\frac{\mathrm{q}}{2k}x^2 + Ax + B \tag{7}$$

with A and B being integration constants. Equation 7 represents a boundary value problem. To solve equation 7 analytically, we need two boundary conditions. We use the following boundary conditions: i) $dT/dx = 0$ at $x = 0$, i.e., zero heat flux through the center of the cell due to symmetry, and ii) $T = T_0$ at $x = L/2$, the temperature at the edge of the battery corresponds to the temperature of the cooling system, where L is the width of the battery.

The first boundary condition yields $A = 0$, and the second one yields $B = T_0 + \frac{\mathrm{q}L^2}{2k}$. Inserting the two boundary conditions in equation 7, we get

$$T = T_0 + \frac{\mathrm{q}}{2k}((L/2)^2 - x^2). \tag{8}$$

Hence, the maximum temperature at $x = 0$ is equal to

$$T_{max} = T_0 + \frac{\mathrm{q}}{2k}\left(\frac{L}{2}\right)^2. \tag{9}$$

The heat generated in the battery is given by

$$\mathrm{q} = \frac{\mathrm{I}^2 \cdot R_{cell}}{d_{cell}} \tag{10}$$

with I (mA·cm^{-2}) being the product of C-rate η (h^{-1}) and areal capacity C (mAh·cm^{-2}). Substituting into equation 9 gives

$$\Delta T = \frac{(\eta \cdot C)^2 \cdot R_{cell}}{d_{cell} \cdot 2 \cdot \mathrm{k}}\left(\frac{L}{2}\right)^2. \tag{11}$$

The difference between the temperature at the center of the cell and the temperature at the cooling channels is hence proportional to L squared, as shown in Figure S8a. The C-rate can further be described by reformulating equation 11 to

$$\eta = \frac{2}{C \cdot L} \cdot \sqrt{\frac{\Delta T \cdot d_{cell} \cdot 2 \cdot \mathrm{k}}{R_{cell}}}. \tag{12}$$

with the average in-plane thermal conductivity of the battery given by

$$\mathrm{k} = \frac{\Sigma_i d_i k_i}{\Sigma_i d_i}. \tag{13}$$

As a result, the temperature is at a maximum at the center of the battery and decays towards the cooling channels, as shown in Figure S8b.

The operating limits imposed by voltage efficiency, critical current density, and thermal constraints for LCO and NMC811 are shown in Figure S11. It can be seen that depending on the cathode thickness, different effects can limit the maximum achievable C-rate of a battery.

Figure S10 shows the specific energy potential of multi-cell thin-film batteries for different cathode and substrate thicknesses as a function of a number of stacked cells. The highest increase in specific energy is achieved within the first 10 cells. Stacking more cells on top of each other has only a limited effect on the specific energy potential. It can also be seen that when a thicker substrate is used, a larger number of cells must be stacked to compensate for the substrate's weight and achieve the same specific energy.

Supplementary Figures

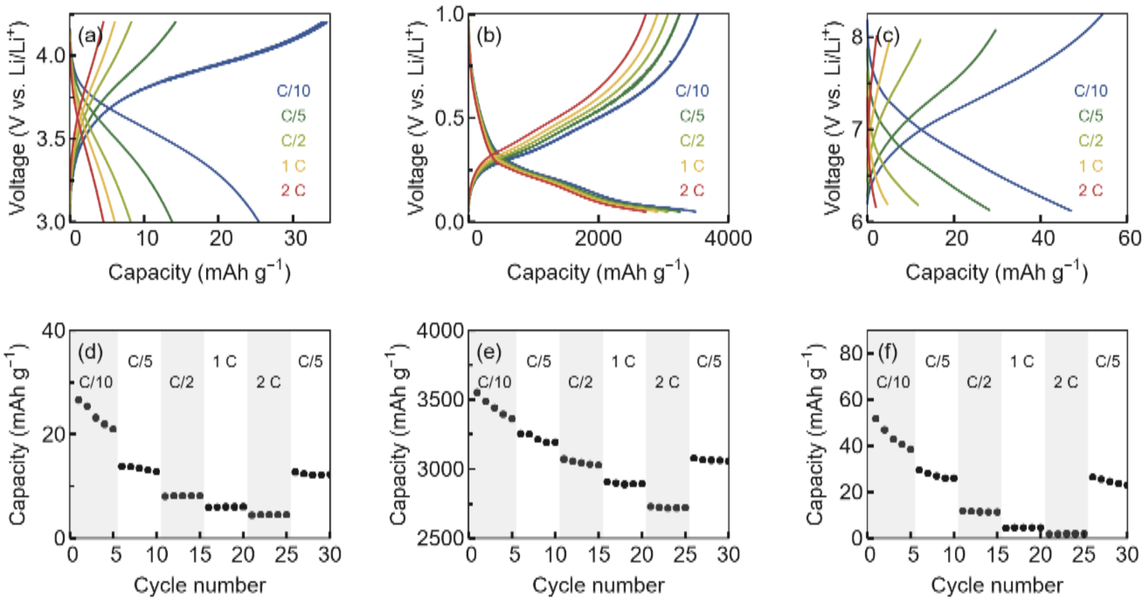

Supplementary Figure S1. Charge-discharge curves and discharge capacity of (a) and (d) LCO/Lipon/Li cell, (b) and (e) Si/Lipon/Li cell, and (c) and (f) multi-cell battery, respectively. The C-rates are calculated assuming a capacity of 70 mAh·g^{-1} for LCO and 4.2 Ah·g^{-1} for Si, resulting in a current density of 10, 49, and 10 μA·cm^{-2} for the LCO cell, the Si cell, and the cell battery at 1 C, respectively.

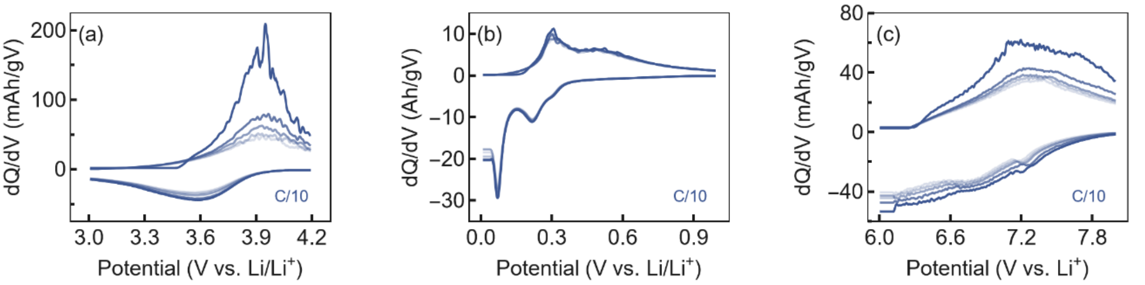

Supplementary Figure S2. Differential capacity profiles of (a) LCO/Lipon/Li cell, (b) Si/Lipon/Li cell, and (c) multi-cell battery over five cycles.

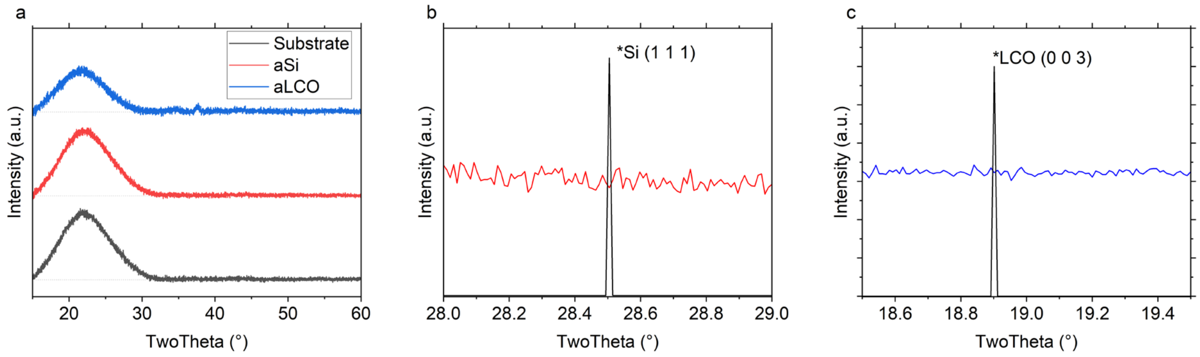

Supplementary Figure S3. (a) Bragg-Brentano XRD diffractograms of as-deposited 300 nm thick Si and LCO on alkaline alumino-borosilicate glass substrates (Corning EXG). (b) Grazing-incidence XRD (GI-XRD) of as-deposited 300 nm thick LCO at an incident angle of 1° in the two theta angle range of 18.5° to 19.5°. (c) GI-XRD of as-deposited 300 nm thick Si at an incident angle of 1.5° in the two theta angle range of 28° to 29°.

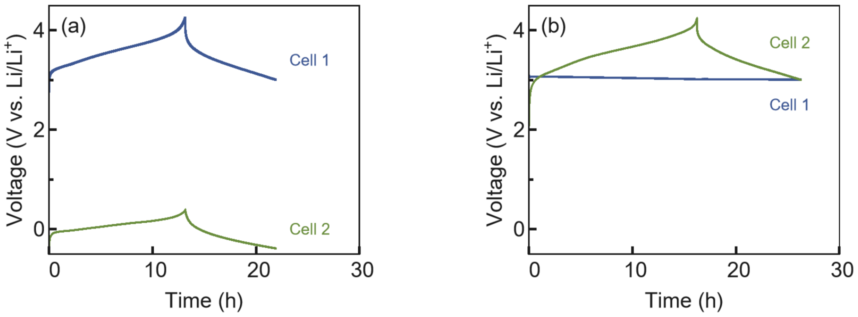

Supplementary Figure S4. Pre-cycling of **(a)** the bottom cell (cell 1) and **(b)** the top cell (cell 2). The initial charging was performed at C/5 (2 μA·cm^{-2}), and the initial discharging at C/10 (1 μA·cm^{-2}). The initial discharge capacity of the top cell (70 mA·g^{-1}) is higher compared to the initial discharge capacity of the bottom cell (61 mA·g^{-1}).

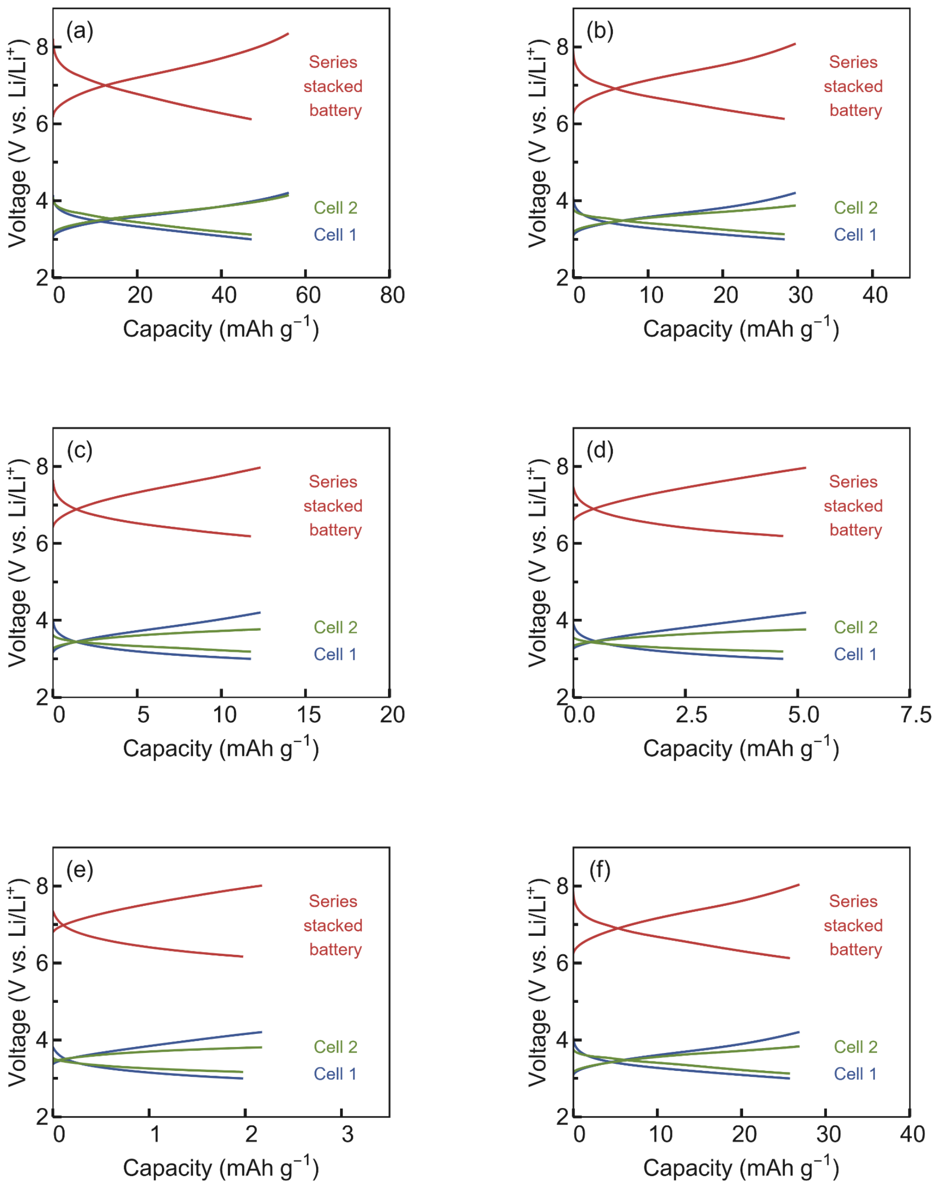

Supplementary Figure S5. Charge–discharge curves of a series-stacked thin-film battery measured at **(a)** C/10 (2 μA·cm^{-2}), **(b)** C/5 (2 μA·cm^{-2}), **(c)** C/2 (5 μA·cm^{-2}), **(d)** 1C (10 μA·cm^{-2}), and **(e)** 2C (20 μA·cm^{-2}), and **(f)** C/5 (2 μA·cm^{-2}). At high C-rates, the voltage difference between cells 1 and 2 becomes greater. The energies of the corresponding charge-discharge curves correspond to the second energy at each C-rate shown in Figure 2e in the main text.

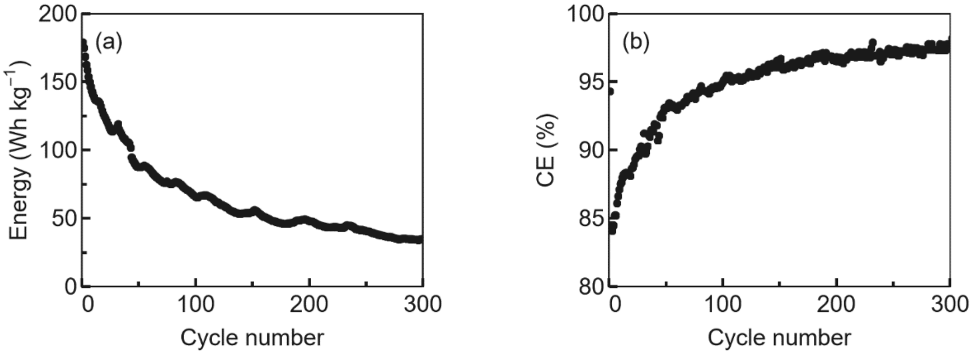

Supplementary Figure S6. (a) Discharge energy and **(b)** coulombic efficiency during long-term cycling of a series-stacked thin-film battery over 300 cycles at a current density of 4.2 µA·cm^{-2} (C-rate of ~ C/2). While the battery's energy fades quickly, we show that the battery can operate for hundreds of cycles without failure. The decline in energy seen here and in Figure 2e has been previously observed in single cells using amorphous LCO,[3] so we expect the energy drop to be reduced using a different cathode material.

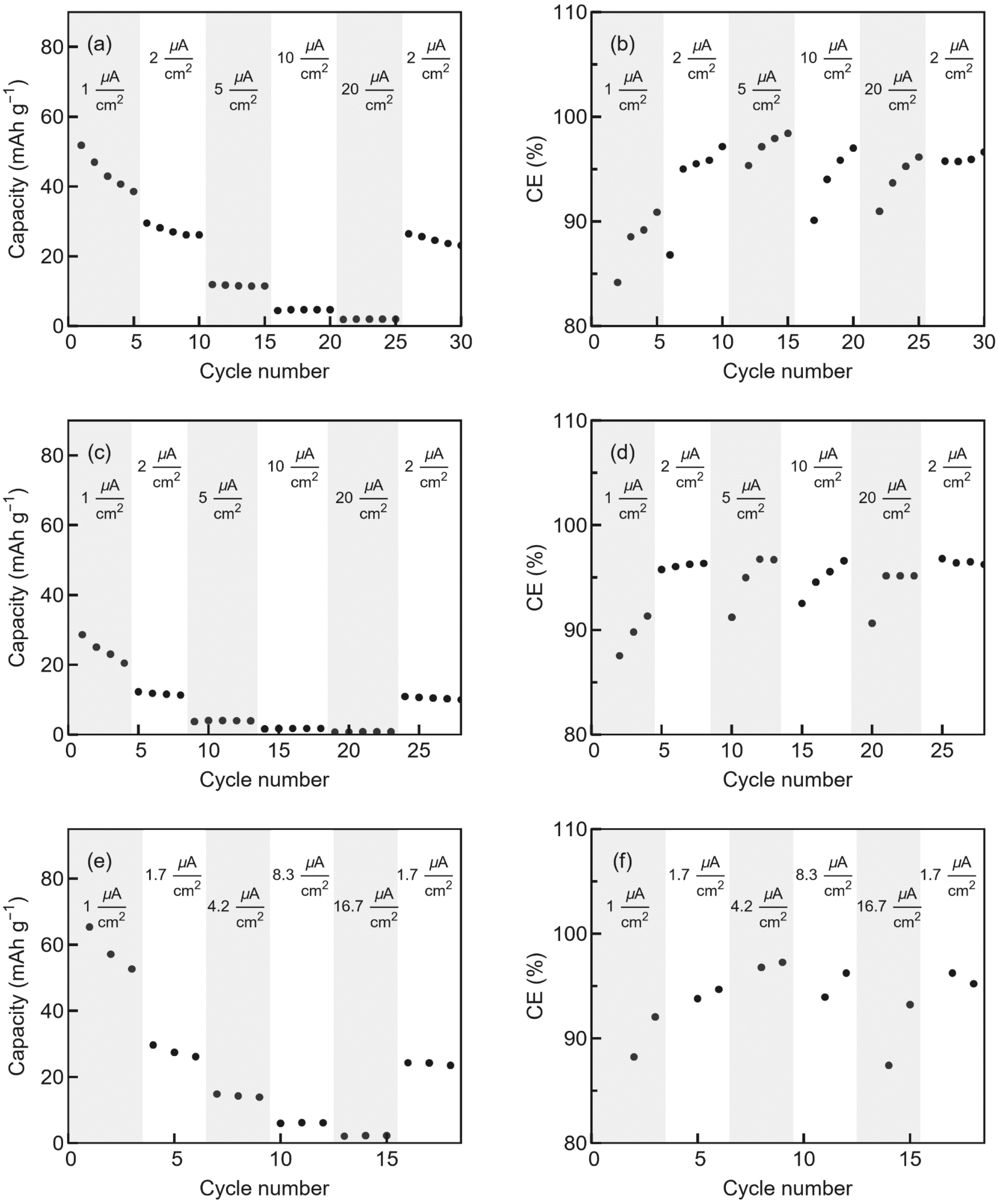

Supplementary Figure S7. Discharge capacity of three individual batteries along with their coulombic efficiencies. **(a,c,e)** Discharge capacity and **(b,d,f)** their respective coulombic efficiency of three series-stacked thin-film batteries, measured at C-rates ranging C/10 (1 μA·cm^{-2}) to 2 C (20 μA·cm^{-2}). The battery shown in a and b corresponds to the battery shown in Figure 2e in the main text. The average capacity across all three batteries at 1 μA·cm^{-2} (C/10) is 42 ± 17 mAh·g^{-1} with an average charge and discharge voltage of 7.17 and 6.56 V, respectively.

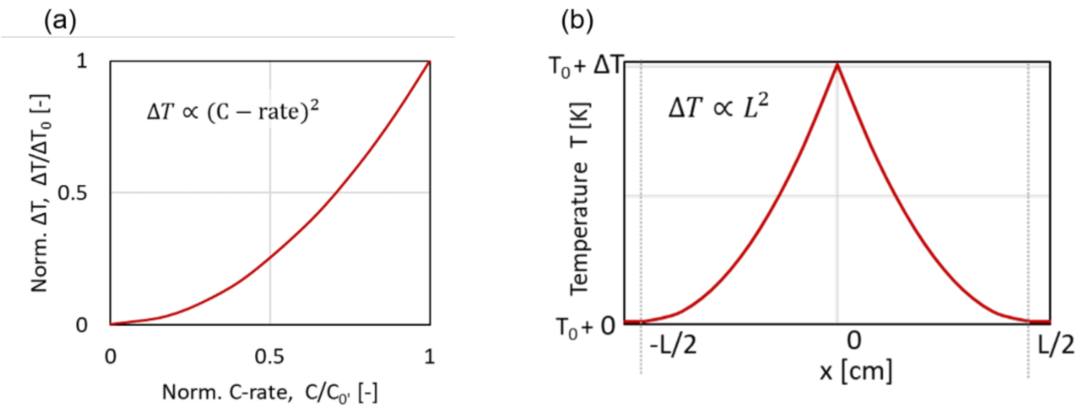

Supplementary Figure S8. **(a)** Temperature difference as a function of C-rate. **(b)** The temperature profile within a battery shows that the temperature is highest in the center of the cell and decreases towards the cooling channels on the side.

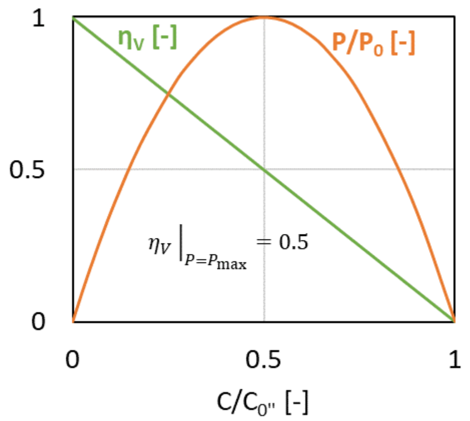

Supplementary Figure S9. Maximum power as a function of C-rate. The maximum power occurs at a voltage efficiency of 0.5.

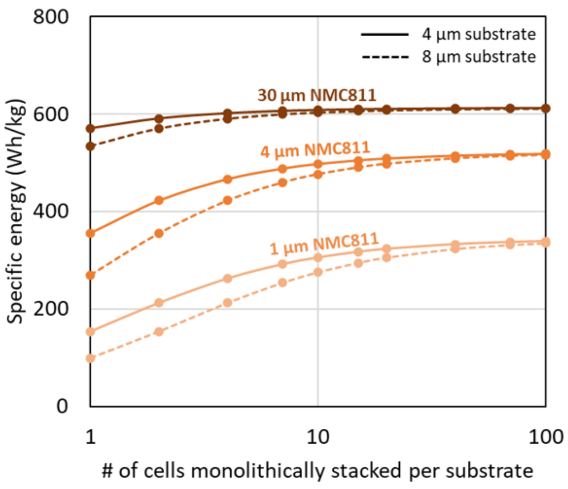

Supplementary Figure S10. The specific energy potential of multi-cell thin-film batteries for different cathode and substrate thicknesses as a function of a number of stacked cells.

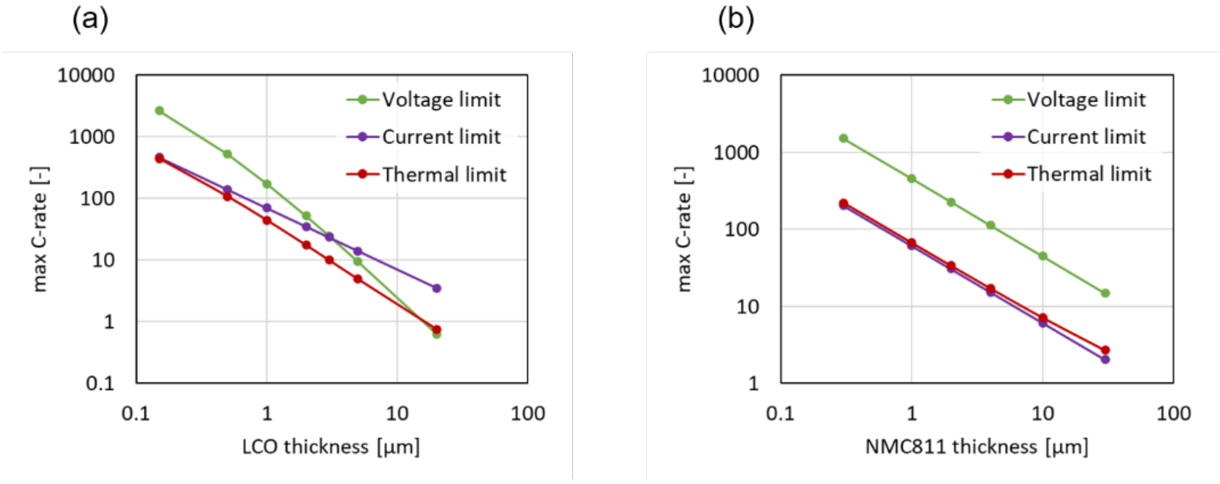

Supplementary Figure S11. Operating limits imposed by voltage efficiency, critical current density, and thermal constraints for (a) LCO and (b) NMC811.

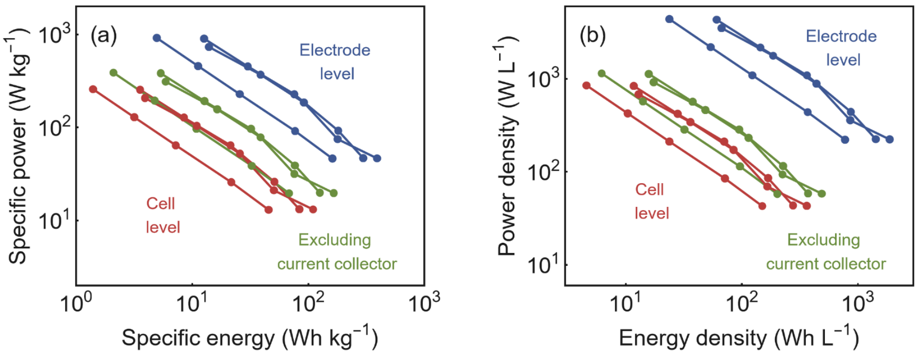

Supplementary Figure S12. Ragone plots of **(a)** the specific (gravimetric) energy and power and **(b)** the volumetric energy and power densities of three individual series-stacked thin-film batteries shown in Figure S7. The blue data points include only the electrode, the green data points exclude the current collectors and substrate, and the red data points exclude the substrate. The calculated energy densities are based only on the cell's active area and neglect the substrate. Al, LCO, Lipon, Si, and Cu densities are assumed to be 2.7, 4.79, 2.3, 2.33, and 8.96 g·cm^{-3}, respectively. The mass of each layer of a cell is 2.4 µg for Al, 4.3 µg for LCO, 5.5 µg for Lipon, 0.3 µg for Si, and 2.7 µg for Cu. The total mass of the battery is 30.6 µg.

Supplementary References

1. Qiu, X.-Y. *et al.* Electrochemical and electronic properties of $LiCoO_2$ cathode investigated by galvanostatic cycling and EIS. *Phys. Chem. Chem. Phys.* **14**, 2617 (2012).
2. Loveridge, M. J. *et al.* Towards high capacity Li-ion batteries based on silicon-graphene composite anodes and sub-micron V-doped $LiFePO_4$ cathodes. *Sci. Rep.* **6**, 37787 (2016).
3. Futscher, M. H. *et al.* Influence of amorphous carbon interlayers on nucleation and early growth of lithium metal at the current collector-solid electrolyte interface. *J. Mater. Chem. A* **10**, 15535–15542 (2022).
4. Wang, S., Yan, M., Li, Y., Vinado, C. & Yang, J. Separating electronic and ionic conductivity in mix-conducting layered lithium transition-metal oxides. *J. Power Sources* **393**, 75–82 (2018).
